# Endobronchial Ultrasonography to Assess and Evaluate Tracheobronchial Tree Invasion in cT4b Esophageal Cancer Patients Treated with Definitive Chemoradiotherapy: Assessment of Resectability

**DOI:** 10.1245/s10434-024-15621-1

**Published:** 2024-07-06

**Authors:** I. L. Defize, E. M. de Groot, O. van de Langerijt, M. van Velzen, S. Mook, N. Haj Mohammad, M. Bulbul, J. P. Ruurda, R. van Hillegersberg

**Affiliations:** 1 Department of Surgery, University Medical Center Utrecht, Utrecht, The Netherlands; 2 Department of Radiation Oncology, University Medical Center Utrecht, Utrecht, The Netherlands; 3 Department of Pulmonary Diseases, University Medical Center Utrecht, Utrecht, The Netherlands; 4 Department of Medical Oncology, University Medical Center Utrecht, Utrecht, The Netherlands

## Abstract

**Background:**

In cT4b esophageal cancer, accurate assessment of tracheobronchial tree invasion after definitive chemoradiotherapy (dCRT) aids in the selection of patients for whom an oncologic radical esophagectomy can be achieved. The current report aimed to determine the accuracy of endobronchial ultrasound in assessing tumor invasion in the tracheobronchial tree after dCRT in patients with cT4b esophageal cancer.

**Methods:**

Esophageal cancer patients with suspicion of tracheobronchial tree invasion on the diagnostic contrast-enhanced computed tomography (CT) who underwent a staging endobronchial ultrasonography (EBUS) were eligible for inclusion in this study. To assess the accuracy of the EBUS in assessing tumor ingrowth in the tracheobronchial tree after dCRT, patients who had an EBUS during restaging and underwent surgery were included in the final analysis.

**Results:**

The final analysis included 26 patients. For 18 (90%) of 20 patients in whom the anatomy of the tracheobronchial tree was restored on the restaging EBUS and tumor invasion was considered to be absent, a radical esophagectomy was achieved. In six patients, persistent ingrowth was observed during the restaging EBUS. For these patients, the EBUS was repeated after a median of 9 weeks. Tumor invasion was considered to be absent in four patients, and a radical resection was achieved in three of these patients.

**Conclusion:**

The EBUS provides valuable information on the assessment of tracheobronchial tree invasion in cT4b esophageal cancer patients after dCRT. This information could aid in the proper selection of patients who benefit from a curative but highly invasive esophagectomy.

The esophagus, situated in the posterior mediastinum, is closely related to the tracheobronchial tree and aorta, facilitating the invasion of esophageal cancer into these structures.^1^ Esophageal cancer patients with tumors that invade the aorta or tracheobronchial tree (cT4b), at diagnosis are often deemed irresectable and treated with definitive chemoradiotherapy (dCRT) alone, precluding them from possible curative surgery.^2^

Recent retrospective reports have shown that patients with cT4b esophageal cancer who undergo dCRT followed by a radical (R0) esophagectomy have better survival than patients who receive dCRT alone.^3–8^ To increase the chance of achieving a radical resection after dCRT in cT4b esophageal cancer, patients should be properly selected during restaging before a curative but highly invasive esophagectomy is performed.^9^

Tracheobronchial tree invasion is observed in up to 80% of cT4b esophageal cancer patients.^10,11^ During restaging, an endobronchial ultrasonography (EBUS) may be considered to assess tumor invasion in the tracheobronchial tree and select patients in whom a radical esophagectomy can be achieved.

Previous studies have reported accurate assessment of tumor invasion in the tracheobronchial tree before neoadjuvant treatment with EBUS.^12–18^ However, structural changes such as fibrosis caused by chemoradiotherapy might impair the ability of an EBUS to determine tracheobronchial tree invasion. Therefore, the current study aimed to determine the accuracy of EBUS in assessing tumor invasion into the tracheobronchial tree after dCRT for patients with cT4b esophageal cancer.

## Methods

### Data Collection and Representation

The data for this study were provided by a prospectively maintained database at the University Medical Center Utrecht. In accordance with the Institutional Review Board, informed consent was waived for the current study. Patient and tumor characteristics were described as counts with percentages, means with standard deviations, or medians with ranges where appropriate using SPSS version 26 (SPSS, Chicago, IL, USA).

### Study Population and Treatment Strategy

Patients with a histopathologically confirmed adenocarcinoma or squamous cell carcinoma of the esophagus with suspicion of tracheobronchial tree invasion of the primary tumor or lymph node metastasis on the diagnostic contrast-enhanced CT who underwent a staging EBUS were eligible for inclusion. To evaluate the accuracy of the EBUS in assessing tumor ingrowth of the tracheobronchial tree after dCRT, patients who had an EBUS acquired during restaging and underwent surgery were included in the final analysis.

### Clinical Staging and Restaging

Clinical staging consisted of an endoscopy with biopsies, an endoscopic ultrasound (EUS), and/or an ^18^F-fluorodeoxuglucose positron emission tomography (PET) and contrast-enhanced computed tomography (CT) of the abdomen, thorax, and neck. An EBUS was acquired for patients with suspicion of tracheobronchial tree invasion on the contrast-enhanced CT.

The criteria for the suspicion of tumor invasion into the tracheobronchial tree comprised dissipation of the fat plane between the esophagus and tracheobronchial tree, indentation on the posterior wall, or signs of displacement of the trachea or bronchus by the tumor.^19^ The results of these diagnostic methods were discussed during a multidisciplinary team (MDT) meeting before the start of any treatment.

To assess the presence of interval metastasis, a PET–CT was acquired 6–8 weeks after completion of dCRT. If no interval metastasis was present and the patient was considered fit for surgery at the MDT meeting, an EBUS was acquired to assess tumor invasion into the tracheobronchial tree. If tumor invasion was absent during the restaging EBUS, the patient proceeded to surgery. Patients with persistent ingrowth and deemed fit for surgery proceeded to a follow-up period, after which another PET–CT and EBUS were acquired. In the absence of tumor ingrowth and distant metastases, these patients proceeded to surgery.

Patients with persistent ingrowth after the follow-up period were considered for thoracic exploration after careful consideration by the MDT and in consultation with the patient. Staging and restaging were performed according to the seventh edition of the American Joint Committee on Cancer (AJCC).^20^

### EBUS

An EBUS was performed with the patient under local anesthesia via lidocaine and sedation with propofol. A linear ultrasound bronchoscope (BF-UC190F; Olympus, Hoofddorp, Noord-Holland) with a wavelength of 12 MHz was used. After oral introduction of the EBUS scope, the trachea, carina, and right and left main bronchus were visualized to assess tracheobronchial displacement, wall destruction, or tumor. Subsequently, the EBUS scope was used to inspect the different layers of the tracheobronchial wall and assess tumor invasion.

### EBUS Evaluation Criteria

**Fig. 1:**
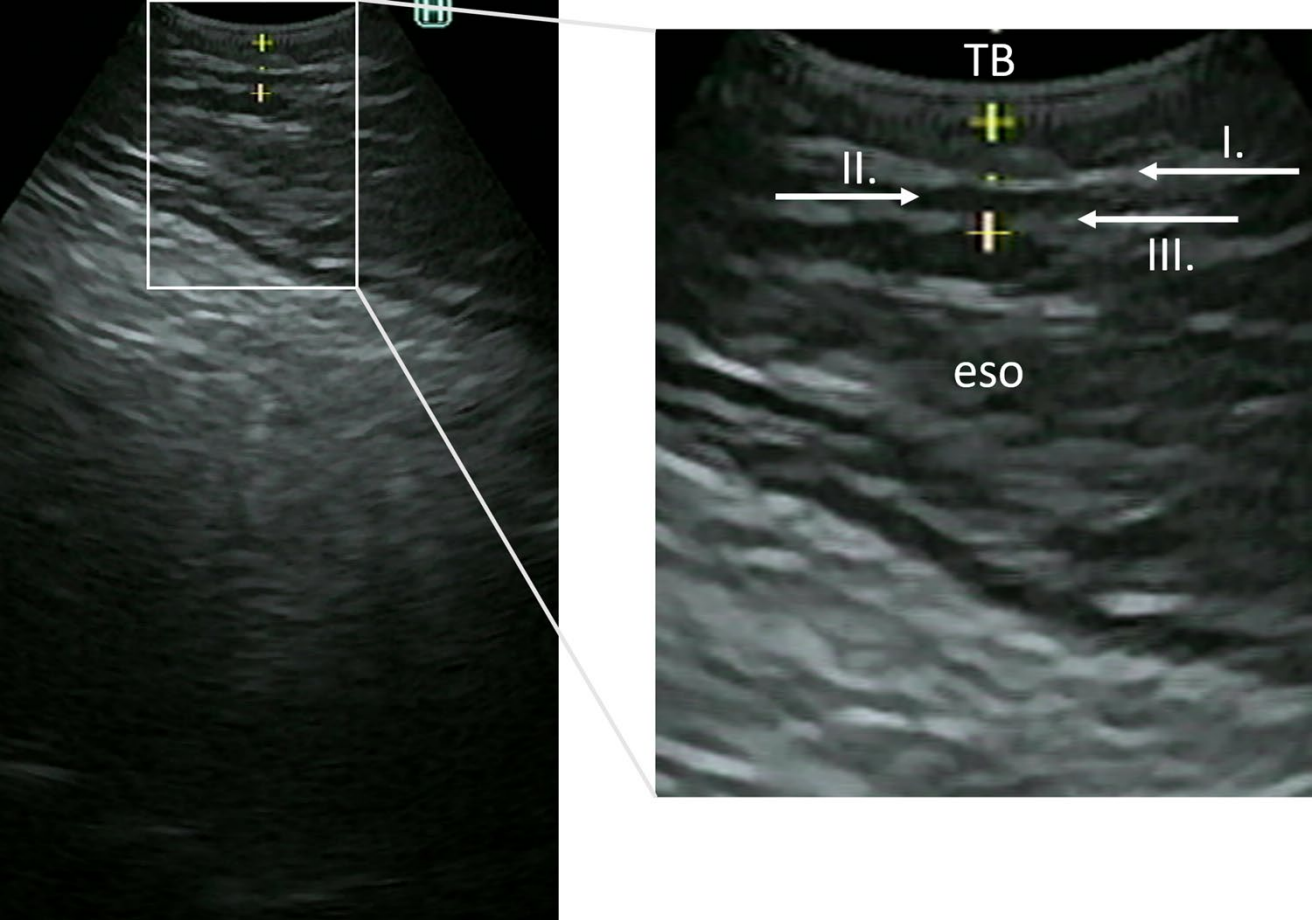
An EBUS image of the tracheobronchial wall as seen in the absence of tumor invasion. Three distinct layers (hyperechogenic, hypoechogenic, and hyperechogenic) are shown. The third layer corresponds with the adventitia of the membranous part of the tracheal wall. EBUS, endobronchial ultrasonography; eso, esophagus; TB, tracheobronchial tree

The EBUS image of the tracheobronchial wall normally shows three layers (hyperechogenic, hypoechogenic, and hyperechogenic layers), as shown in Fig. 1. The third layer corresponds with the adventitia of the membranous part of the tracheobronchial wall.^13^ In case of interruption or absence of this layer, tumor invasion was considered to be present. If this layer was seen continuously, the adventitia was considered intact, and tumor invasion was considered to be absent. The restaging EBUS was evaluated according to the same criteria.

### Definitive Chemoradiotherapy

Definitive chemoradiotherapy was administered according to an extended CROSS regimen consisting of six weekly cycles of intravenous carboplatin (area under the curve [AUC], 2 mg/ml/min) and paclitaxel (50 mg/m^2^) with concurrent radiation therapy (50.4 Gy in 28 fractions).^21^ Volumetric arc therapy based on three- or four-dimensional CT planning was used for treatment and delivery.

### Surgical Procedure

Patients who proceeded to surgery underwent a robot-assisted minimally invasive esophagectomy (RAMIE) with a two-field lymphadenectomy, as described previously.^11^ The robotic technique allowed for meticulous dissection along the vital mediastinal structures using the 10-times-enlarged three-dimensional images and articulating instruments. The monopolar hook was used for the step-by-step dissection of the T4b area. Either an Ivor-Lewis or McKeown procedure was performed, during which the gastric conduit was pulled up through the posterior mediastinal route. The esophagectomy was not combined with the (partial) resection of adjacent structures such as the tracheobronchial tree or aorta.

### Margin Status

To assess the accuracy of the EBUS in the assessment of tracheobronchial tree invasion after dCRT, the margin status of R0 (no margins involved) or R1 (resection margin with macro- or microscopic tumor residual) from the pathology report of the resection specimen was used.^22^ Pathologic assessment of the resection specimen was performed according to the College of American Pathologists.

## Results

### Study Population

Between October 2011 and March 2021, 65 patients were eligible for inclusion in the study. For 12 (18%) patients with suspicion of tracheobronchial ingrowth on the diagnostic contrast-enhanced CT, the staging EBUS did not confirm this. Of these 12 patients, 2 had suspicion of ingrowth in the aorta based on EUS and were excluded from the final analysis. The remaining 10 patients proceeded to neoadjuvant chemoradiotherapy according to the CROSS regimen. In two patients, interval metastasis was observed during restaging, and they were omitted from surgery. A radical resection was achieved for seven of the eight patients who proceeded to surgery.

**Fig. 2:**
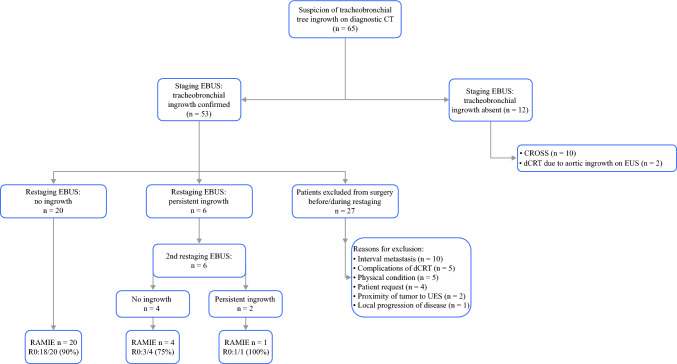
A flowchart of the inclusion and exclusion process. dCRT, definitive chemoradiotherapy; EBUS, endobronchial ultrasonography; EUS, endoscopic ultrasonography; RAMIE, robot-assisted minimally invasive esophagectomy; UES, upper esophageal sphincter; R0, radical resection

In 53 patients, invasion of the tracheobronchial tree was confirmed with the EBUS. The study included 26 patients for the final analysis. The reasons for excluding patients from the final analysis included interval metastasis (*n *= 10), complications of dCRT (*n *= 5), physical condition of the patient (*n *= 5), patient’s request (*n *= 4), proximity of the primary tumor to the upper esophageal sphincter impeding surgical reconstruction (*n *= 2), and local progression of disease with encasement of the left atrium and aorta (*n *= 1). A flowchart of the inclusion and exclusion process is provided in Fig. 2, Table 1 presents the patient and tumor characteristics of the included patients.

**Table 1:** Baseline patient demographics and tumor characteristics of included patients

Characteristic	*n*	%
	Total	
	26	
Sex		
Male	19	73
Female	7	27
Mean age: years (±SD)	64	±7.7
Mean BMI: kg/m^2^ (±SD)	23	±3.4
ASA classification		
1	1	4
2	19	73
3	6	23
Histopathology^a^		
Adenocarcinoma	0	N/A
Squamous cell carcinoma	26	100
metTumor location^b^		
Upper 1/3	11	42
Middle 1/3	15	58
Origin of invasion		
Primary tumor	25	96
Metastatic lymp node	1	4
Location of tumor invasion on EBUS		
Trachea	16	62
Carina	3	12
Left main bronchus	7	27
Right main bronchus	0	N/A
Clinical N stage		
N0	3	11
N1	14	54
N2	7	27
N3	2	8
Surgical procedure		
Ivor-Lewis	3	12
McKeown	22	85
No surgery	1	3

SD, standard deviation; BMI, body mass index; ASA, American Society of Anesthesiologists; EBUS, endobronchial ultrasonography; N/A, not applicable

^a^Determined in pre-treatment biopsy. ^b^Determined at pre-treatment endoscopy

### Tracheobronchial Tree Invasion Absent After dCRT

**Fig. 3:**
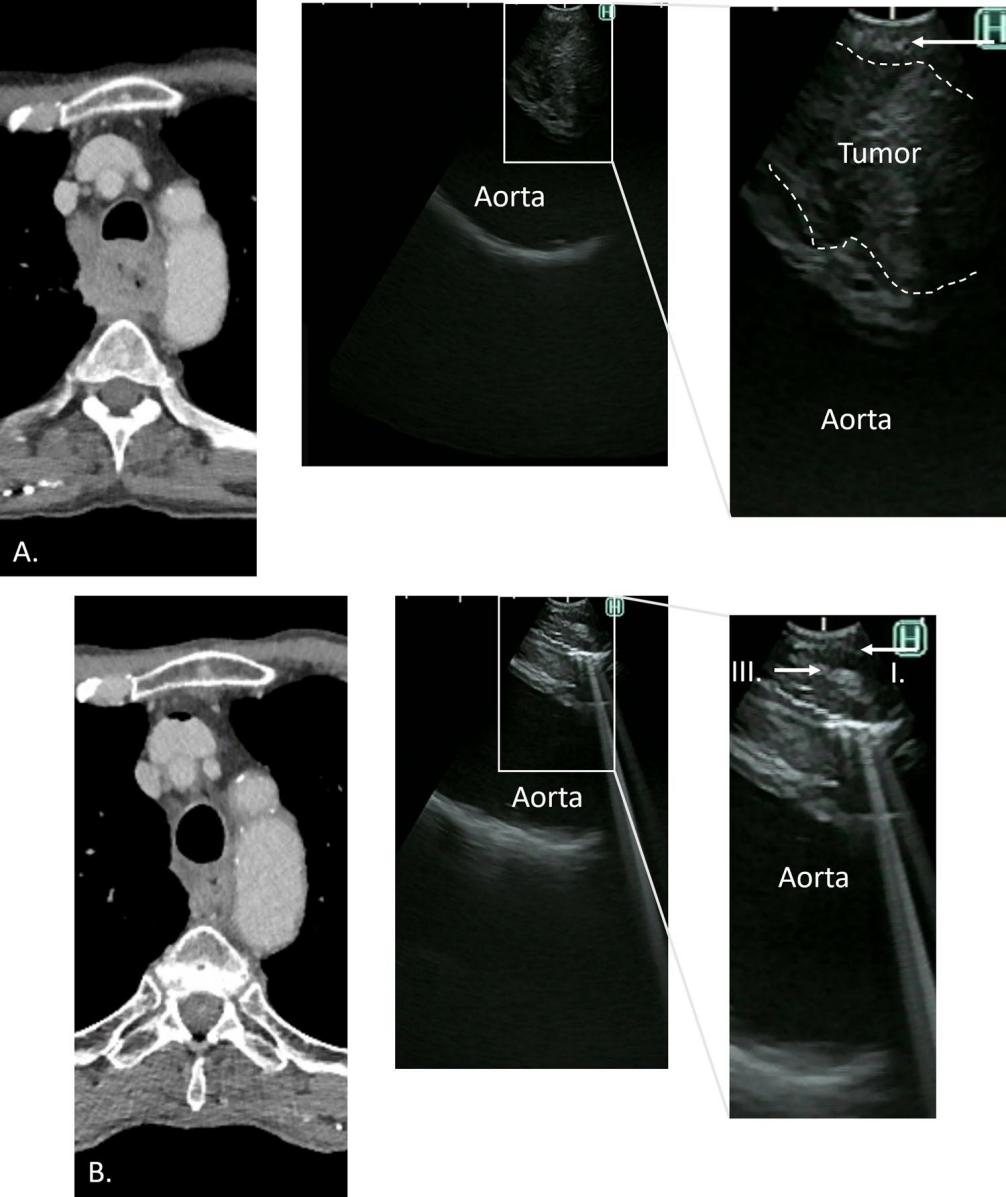
**A** A computed tomography CT (left) image of a patient with suspicion of tumor invasion into the tracheabronchial wall before dCRT. On the EBUS (right), the adventitia (third layer) is not recognizable, and tumor invasion was considered to be present. **B** A CT (left) and EBUS (right) image of the same patient after dCRT in which the third layer is recognizable on the EBUS, and tumor invasion was considered to be absent. EBUS, endobronchial ultrasonography

For 20 patients, the tracheobronchial tree invasion present before dCRT was considered to be absent during the restaging EBUS, and surgical resection was performed. A radical resection was achieved in 18 (90%) of the 20 patients. The median time between completion of dCRT and the restaging EBUS was 7 weeks (range, 2–12 weeks). The median interval between completion of dCRT and surgery was 13 weeks (range, 6–22 weeks). Figure 3A exhibits the image of a patient with tracheobronchial tree invasion before dCRT and restoration of the anatomic layers of the esophagus and trachea after dCRT, as seen on the EBUS and CT (Fig. 3B).

### Persistent Tracheobronchial Tree Invasion After dCRT

In six patients, persistent tracheobronchial tree invasion was considered to be present during the restaging EBUS. In these patients, the EBUS was repeated after a median of 9 weeks (range, 6–22 weeks). Ingrowth was considered to be absent on the subsequent EBUS in four patients, and persistent ingrowth was observed in two patients. In three of four patients with absent tumor invasion at the second EBUS, a radical resection was achieved. In one patient with persistent ingrowth, a thoracic exploration was performed after careful consideration of the multidisciplinary team and consultation with the patient, which resulted in a radical (R0) resection. In the other patient with persistent ingrowth, a thoracic exploration was considered futile due to the extent of ingrowth observed.

**Fig. 4:**
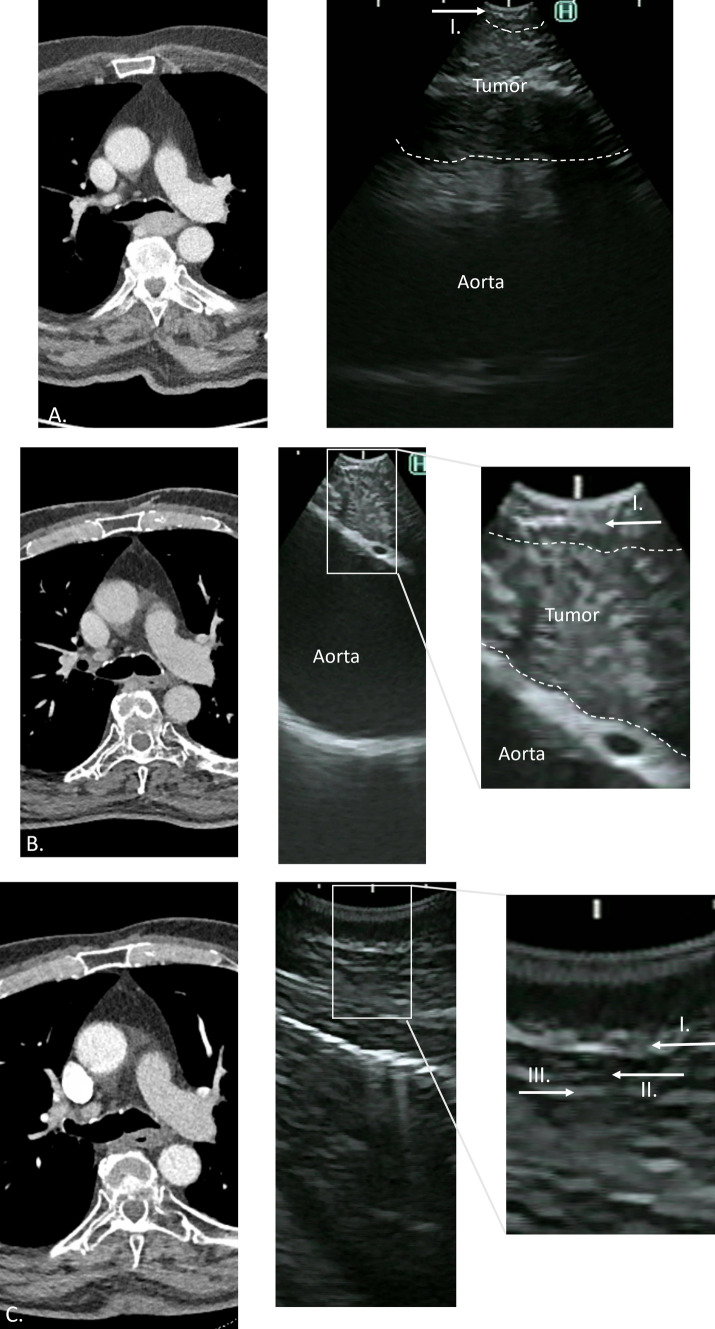
**A** A CT (left) image of a patient with suspicion of tumor invasion into the tracheabronchial tree before dCRT. On the EBUS (right) image, only the first of the three layers is recognizable on the EBUS, and tumor invasion in the tracheobronchial tree was considered to be present. **B** A CT (left) image of the same patient after dCRT. The EBUS (right) image shows an intact first layer, and tumor regression was observed. However, the adventitia (third layer) is absent, and persistent tumor invasion was considered to be present. **C** A CT (left) image of the same patient after an interval of 7 weeks. On the EBUS (right) image, the three layers are recognizable, and tumor invasion was considered to be absent. CT, computed tomography; dCRT, definitive chemoradiotherapy; EBUS, endobronchial ultrasonography

Figure 4A, provides an image of a patient with tracheobronchial invasion before dCRT. Figure 4B shows an image of the same patient with persistent tumor invasion at restaging. The EBUS acquired during follow-up evaluation showed the dissipation of tumor invasion and restoration of the normal anatomy (Fig. 4C).

## Discussion

A radical resection was achieved for 90% of the patients whose tracheobronchial tree invasion was considered to be absent during the restaging EBUS. This demonstrates that the EBUS provides valuable information in the assessment of tracheobronchial tree invasion for cT4b esophageal cancer patients after dCRT. This information could aid in the proper selection of cT4b esophageal cancer patients who might benefit from a curative esophagectomy after dCRT.

Currently, most cT4b patients are precluded from surgery at diagnosis regardless of the response of the primary tumor to neoadjuvant treatment.^2^ Favorable results in terms of survival have been demonstrated for patients who undergo an esophagectomy after dCRT.^8,11,23,24^ However, these results are largely dependent on the radicality of the resection because an incomplete resection often results in a dismal survival.^7^ Furthermore, the surgical exploration of a tumor with persistent tracheobronchial ingrowth may cause serious damage to the membranous part in irradiated tissue. Therefore, patients should be properly selected based on the feasibility of an oncologic radical and safe procedure.

Previous reports have shown that significant downstaging in terms of tumor volume regression occurs during chemoradiotherapy.^25^ The current report demonstrates that tumor volume regression might lead to the dissipation of tumor invasion and restoration of the normal anatomy, which can be observed by an EBUS, aiding in the determination of resectability.

In our experience, the restored adventitia was characterized by a continuous line with hyperechoic dashes, differing from an unaffected adventitia, indicating the dissipation of tumor invasion. Additionally, it was previously hypothesized that treatment-induced changes such as fibrosis might impair the ability of an EBUS to determine tracheobronchial tree invasion.^26,27^ The current report demonstrates that the anatomic borders of the esophagus and the tracheobronchial tree can be recognized by EBUS after dCRT, allowing the assessment of resectability.

The current report demonstrates that the effects of chemoradiotherapy persist well after restaging. In four patients who proceeded to follow-up evaluation due to persistent ingrowth at restaging, tumor invasion had dissipated during the second EBUS, and a radical resection was achieved in three of these patients. This finding might act as an incentive to actively monitor patients with persistent ingrowth during follow-up evaluation with an EBUS.

The current study had some limitations. First, the current results are limited to the negative predictive value of the EBUS because the study analyzed data from real-world clinical practice in which patients with persistent ingrowth were precluded from surgery. Therefore, it was not possible to objectify all diagnostic characteristics such as the sensitivity and specificity of the EBUS.

Another limitation was the retrospective and heterogeneous nature of the current data, which might reduce the level of evidence of the current results. Nevertheless, to date, this is the largest series of cT4b patients in which the diagnostic performance of the EBUS after dCRT was assessed. Therefore, these results should act as an incentive to incorporate the EBUS into future prospective studies.

The acquisition and interpretation of EBUS images require an experienced pulmonologist sonographer and are subject to inter- and intra-observer variability, which might complicate their clinical implementation. These challenges should not impede future research into this method in cT4b esophageal cancer because conventional methods such as PET–CT, which lack sufficient resolution to assess the local status of a tumor, are being used to exclude patients from possible curative surgery. The EBUS provides an accurate assessment of the local status of esophageal cancer, enabling the assessment of resectability, which is pivotal in the curative treatment of cT4b esophageal cancer.

In conclusion, this is the first study to assess the ability of EBUS to assess tracheobronchial tree invasion after dCRT in cT4b esophageal cancer. Our results demonstrate the valuable information provided by the EBUS on local tumor status and resectability. The current results should act as an incentive to explore the application of this diagnostic method in the selection of cT4b esophageal cancer patients who might benefit from curative surgery after dCRT.
